# Low Level Sequence Variant Analysis of Recombinant Proteins: An Optimized Approach

**DOI:** 10.1371/journal.pone.0040328

**Published:** 2012-07-06

**Authors:** Anne Zeck, Jörg Thomas Regula, Vincent Larraillet, Björn Mautz, Oliver Popp, Ulrich Göpfert, Frank Wiegeshoff, Ulrike E. E. Vollertsen, Ingo H. Gorr, Hans Koll, Apollon Papadimitriou

**Affiliations:** Biologics Research, Pharma Research and Early Development, Roche Diagnostics GmbH, Penzberg, Germany; Technical University of Braunschweig, Germany

## Abstract

Sequence variants in recombinant biopharmaceuticals may have a relevant and unpredictable impact on clinical safety and efficacy. Hence, their sensitive analysis is important throughout bioprocess development. The two stage analytical approach presented here provides a quick multi clone comparison of candidate production cell lines as a first stage, followed by an in-depth analysis including identification and quantitation of aberrant sequence variants of selected clones as a second stage. We show that the differential analysis is a suitable tool for sensitive and fast batch to batch comparison of recombinant proteins. The optimized approach allows for detection of not only single amino acid substitutions in unmodified peptides, but also substitutions in posttranslational modified peptides such as glycopeptides, for detection of truncated or elongated sequence variants as well as double amino acid substitutions or substitution with amino acid structural isomers within one peptide. In two case studies we were able to detect sequence variants of different origin down to a sub percentage level. One of the sequence variants (Thr → Asn) could be correlated to a cytosine to adenine substitution at DNA( desoxyribonucleic acid) level. In the second case we were able to correlate the sub percentage substitution (Phe → Tyr) to amino acid limitation in the chemically defined fermentation medium.

## Introduction

Monoclonal antibodies have become a well established and fast growing class of biotherapeutics [1], [2]. They have been approved for the treatment of diseases such as cancer, cardiovascular diseases, inflammatory, infectious and autoimmune diseases [3], [4]. Stably transfected cell lines derived from CHO (Chinese hamster ovary), NS0, Sp2/0 or other mammalian cells are now widely used to produce therapeutic monoclonal antibodies in high amounts [4]–[6]. To achieve the required product titer, the methodologies for cell line development, cell culturing and down stream processing have been optimized [7], [8]. Furthermore, the quality of the biotherapeutics needs to be closely monitored to ensure product efficacy and safety. Product quality attributes such as structural integrity, aggregation, charge heterogeneity, glycosylation pattern or amino acid degradation are analyzed using a variety of different analytical methods.

One of the most challenging analytical methodologies used to ensure product quality is the sensitive and comprehensive detection of sequence variants. During the last years, unintended amino acid substitutions have been reported in recombinant proteins expressed in mammalian cell culture. Some of these misincorporations were shown to be due to DNA mutations. A Tyr→Gln variant form of an antibody was found to be produced by a subpopulation of transfected Chinese hamster ovary (CHO) cells bearing point mutations in the heavy chain gene [9]. In a more recent study, CHO subclones exhibited a Phe → Leu misincorporation in a recombinant peptide-antibody fusion protein originating probably from partially mutated gene copies introduced into the cells [10]. Another study reports the replacement of serine by arginine due to a DNA point mutation, and correlates the mutation rate positively with the methotrexate (MTX) concentration used for stable cell line selection and amplification [11].

More recently, misincorporations have been reported to occur during the translation step in protein synthesis, and were referred to as mistranslations. Starvation of cells due to the limitation of amino acids in the fermentation media have been reported to lead to misincorporation of serine at asparagine positions in recombinant antibodies expressed in CHO cells [12], [13]. A codon-specific serine to asparagine mistranslation has been reported by Yu et al. [14] for the serine codon AGC. Mischarging of tRNAs by aminoacyl-tRNA synthetases or misreading of codons due to codon-anticodon mispairing are being discussed as the underlying mechanism of mistranslation [11]–[14].

**Figure 1:**
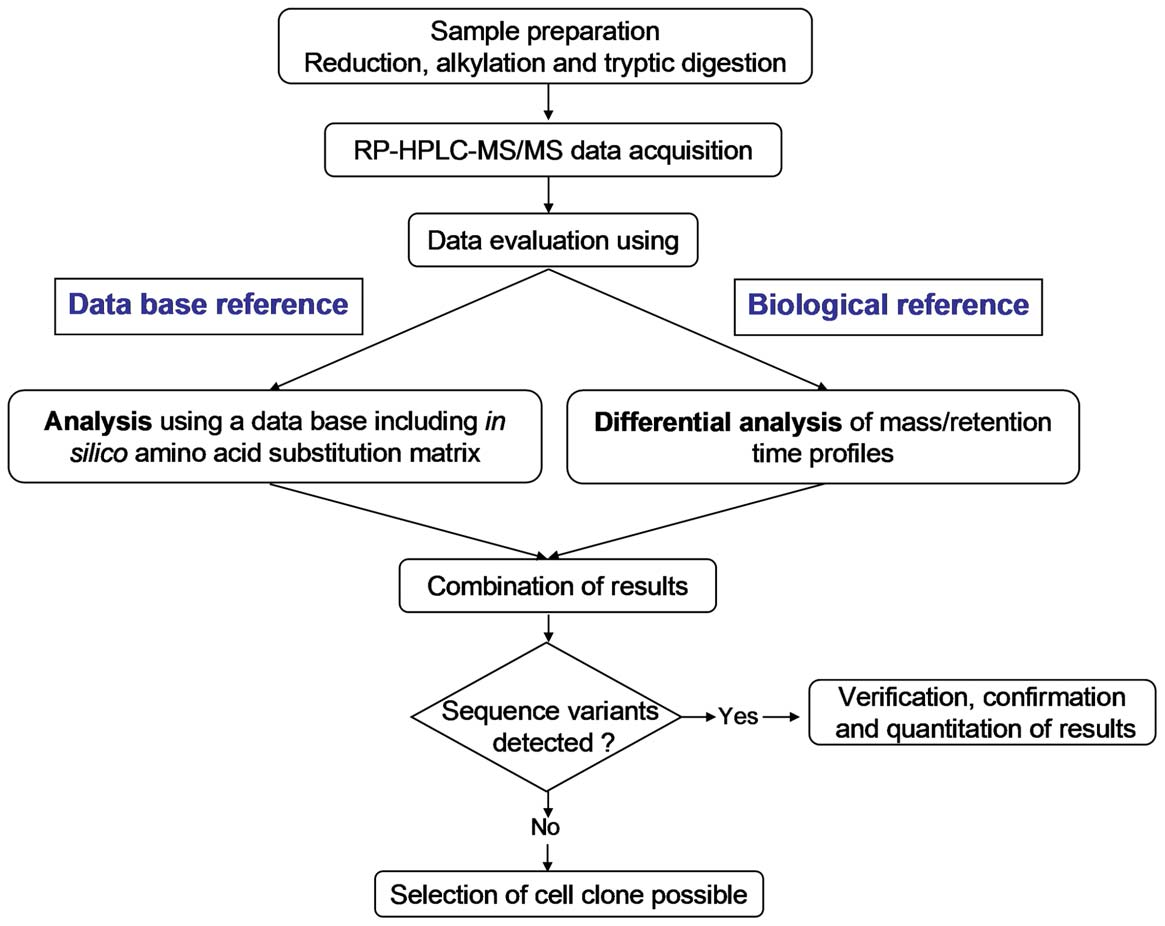
Workflow of sequence variant analysis of recombinant monoclonal antibodies.

As the level of amino acid misincorporation can increase with cell age [9], it is essential to detect even very low levels of sequence variants in the early stage of the cell line generation process. Advanced mass spectrometry technology and related data analysis software tools are capable to fulfill these requirements. Recently, a procedure was described that detects and identifies sequence variants in recombinant human monoclonal antibodies (rhumAb(s)) by combining HPLC-UV/MS/MS characterization of peptide maps with a Mascot based error tolerant search (ETS) [15], [16]. The ETS was introduced by Creasy and Cottrell for database matching of uninterpreted tandem MS data [17]. In addition to a list of common chemical and post-translational modifications, amino acid substitutions were added that can result from single base substitutions within the corresponding codons. The ETS mode has been implemented in the commercially available Mascot web-based computer search program.

**Table 1:** Tryptic peptides unique to rhumAb B identified by Mascot ETS and SIEVE, respectively, at 1% spiking level.

#	# of trypticpeptide	# of AA	MH+	ID byMascot	ID bySIEVE
1	HC T14	49	5056.55	+	+
2	HC T21	33	3789.76	+	+
3	LC T6	30	3363.50	+	+
4	HC T11	29	3160.42	+	+
5	HC T19	27	2971.40	+	+
6	HC T38*	23	2803.24	+	+
7	LC T3	21	2352.17	+	(+)
8	HC T2	19	2249.97	+	+
9	HC T4	21	2197.12	+	+
10	HC T1	20	1910.05	+	+
11	HC T35*	17	1901.93	+	+
12	LC T1	18	1898.02	+	+
13	HC T32	16	1876.90	−	+
14	HC T15	14	1665.75	+	+
15	HC T13**	14	1424.69	+	+
16	HC T8	11	1338.68	+	+
17	HC T12**	12	1288.64	+	+
18	LC T7	12	1264.63	+	+
19	HC T9	11	1247.54	+	+
20	HC T23*glycosylated	9	1173.52	−	+
21	LC T4**	9	1064.57	+	+
22	HC T28	8	830.46	(+)	+
23	LC T2	6	708.33	+	+
24	LC T5	7	685.40	−	−
25	HC T6	5	623.35	+	+
26	HC T3	5	504.28	−	−
27	HC T22	4	501.31	−	−
28	LC T8	4	488.31	−	−
29	HC T18	4	462.26	−	−
30	HC T7	4	461.24	−	−
31	HC T16	3	361.21	−	−
32	HC T5	2	232.14	−	−
33	HC T10	2	232.14	−	−
34	HC T17	1	175.12	−	−

Positive identification by SIEVE is given for ratio values >2.5; positive identification by Mascot is given for ion score values >20 using a database that contains the spiking antibody sequence.

* Tryptic peptide differs in one amino acid between rhumAb A and rhumAb B.

** Tryptic peptide differs in two amino acids between rhumAb A and rhumAb B.

(+)These peptides gave a slightly lower score in SIEVE and Mascot respectively but still were considered as identified after manual data interpretation.

**Figure 2:**
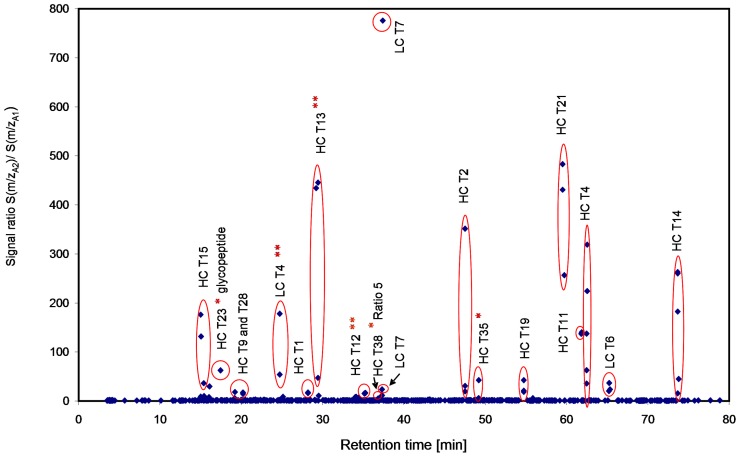
Scatter plot showing differences in m/z and retention time domain between the LC-MS/MS data sets of rhumAb A tryptic digests with ant without 1% rhumAb B. For labeling nomenclature see Table 1.

Even though this data analysis package pioneered the sensitive detection of sequence variants in recombinant proteins it has certain limitations. One major draw-back is the relatively high number of false positive matches. For example, oxidation of amino acids, e.g. methionine, is isobaric to Phe → Tyr or Ala → Ser substitution and carboxymethylation of amino acids is isobaric to Ala → Glu or Gly → Asp substitution. In order to assign those matches correctly, manual data evaluation and expertise in the chromatographic retention time and mass spectrometric fragmentation behavior of the modified peptides are necessary. Furthermore, not all sequence variants are covered by the error tolerant search. Double mutations or certain variants resulting from mischarging of the tRNA might be missed.

To overcome these limitations of the Mascot ETS based evaluation we added a second data analysis approach. In contrast to the Mascot ETS analysis that is based upon theoretical sequences as “reference”, we use biological samples as reference analyzing two or more LC-MS/MS data sets in a differential way. For this purpose, we use the commercially available SIEVE software. It was originally designed for label-free quantitative differential expression analysis of proteins and peptides [18]. The software first aligns the chromatographic pattern based on retention time of the two samples and then compares them incrementally by using “frames” defined by retention time and m/z windows. A specific ratio value is calculated for corresponding peak pairs within one frame. The ratio value is equal to 1 if no change in signal intensity is observed. If new peaks belonging to e.g. sequence variants appear, the ratio values are defined as the signal intensities of the new peaks and they are therefore detected easily. The combination of Mascot ETS and SIEVE analysis being two complementary data evaluation workflows increases the likelihood, speed and confidence for detection of sequence variants while keeping the additional experimental effort limited (Figure 1).

**Table 2:** Assignment of hits detected by differential analysis (see Figure 4) and relative quantitation of single amino acid mutations.

#	RT [min]	SIEVE ratio value^1^	MH+	Mascot ID	Manual ID (rel. amount at 2L/100 L fermentation scale [%])	Comment
1	14.7	10	3440.46	−	HC T22-23+biantGal2wFuc^2^	production clone specific glycopattern differences
2	15.7	11	2958.16	−	HC T22+biantGal2wFuc	
3	18.1	22	2121.95	+	LC T14 **Glu→Asp** (0.4/0.5)	single point mutation
4	22.7	6	1876.92	+	LC T18	unmodified tryptic peptide
5	36.8	7	2574.29	−	TATGVHS-LC T1 (0.7/n.d.)	production clone specific signal peptide remaining^3^
6	43.0	7	1920.97	+	LC T1	unmodified tryptic peptide
7	55.1	312	1821.01	+	HC T24 **Thr→Asn** (2.7/4.0)	single point mutation^3^
8	58.7	172	1803.98	−	Not identified	

1 :Highest Sieve ratio value found for the respective peptide.

2 :biantGal2wFuc: core fucosylated, fully galactosylated complex biantennary glycostructure.

3 :for assignment of the corresponding MS/MS spectrum see Figures S2 and S3.

## Results

### Method Optimization and Assessment of Critical Method Parameters: Sequence Coverage, Sensitivity and Quantification

In order to assess the critical method parameters we analyzed a tryptic digest of rhumAb A spiked with 1% (v/v) rhumAb B. The two antibodies were selected with regard to a maximum number of unique tryptic peptides. The *in-silico* tryptic digest of the two antibodies revealed 34 unique peptides, including 9 small peptides with molecular masses less than 600 Da present in rhumAb B but not in rhumAb A (Table 1). Among the unique peptides there were three peptides differing in only one amino acid, including the glycopeptide, and three unique peptides differing in two amino acids between antibody B and antibody A. All other tryptic peptides differed in more than two amino acids. In order to compare the sensitivity of both data evaluation approaches we added the spiked antibody sequence to our in-house build protein database and used the regular Mascot database search. The identification of the unique peptides by Mascot and by Sieve, respectively, is summarized in Table 1. The unique peptides spiked at 1% level were identified with both data evaluation approaches down to *m/z* values of approx. 600 amu with high confidence. The glycopeptide as well as the proline-rich heavy chain (HC) peptide HC T32, which gave a poor ion score in the Mascot ETS, was only identified by SIEVE. The sequence coverage of the unique amino acids is therefore 87% using Mascot data base search and 92% using the SIEVE based data analysis approach.

**Figure 3:**
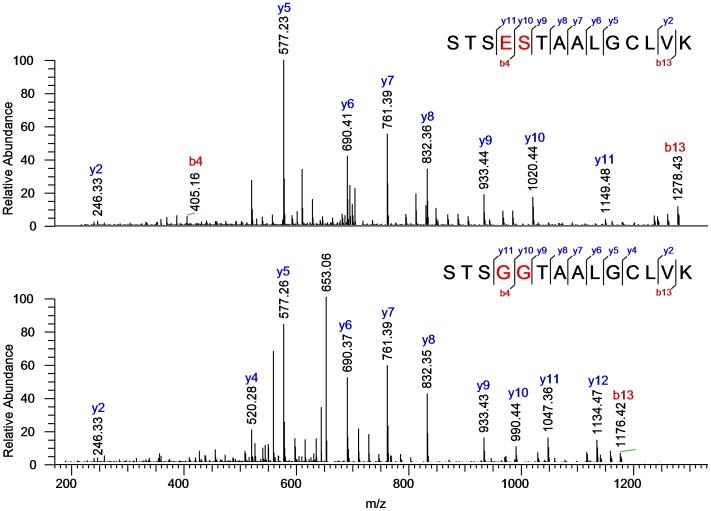
Fragment ion spectra of the HC T13** peptide originating from the spiking antibody B (upper spectrum) and the corresponding peptide originating from the reference antibody A (lower spectrum). SIEVE detected the HC T13 peptide and MS/MS was triggered.

By visual comparison of the total ion chromatograms (TICs) and the UV traces at 220 nm none of the unique spiked peptides were detected (data not shown). However, differences at the level of 1% between the two samples became visible when using both, the chromatographic retention time and the exact mass (m/z) for comparison using the SIEVE software. Figure 2 shows the corresponding scatter plot and demonstrates that the unique spiked peptides with one and two amino acid differences, including the glycopeptide, clearly emerged as differential signals. If different charge states for one and the same peptide were identified in individual frames, a single unique peptide can appear several times in the scatter plot.

We further simulated the combination of the results obtained by the two data evaluation approaches and tried to identify signals found by differential analysis but not by data base search. For this purpose we used the fragment ion spectra of peptides unassigned by data base search and searched for identical mass fragment series or delta mass series in one of the identified peptides’ MS/MS spectra. As an example, the identification of peptide HC T13** is demonstrated in Figure 3. The series of y-ions from y5 until y9 clearly assigns the modified peptide to the unmodified peptide containing the “TAALG” or reverse sequence tag and therefore helped to “manually” identify the doubly mutated peptide that was missed by the Mascot ETS.

**Figure 4:**
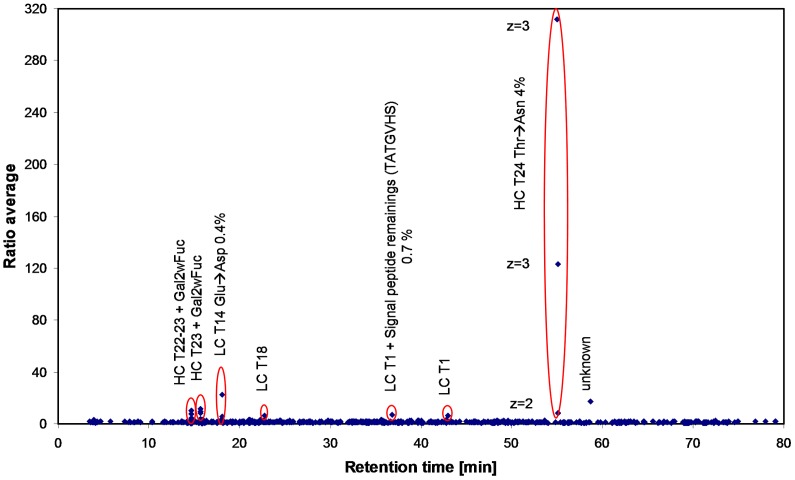
Scatter plot showing differences in m/z and retention time domain of two LC-MS/MS data set replicates from tryptic digests of recombinant human IgG1 antibody A clone 1 and clone 2.

In a last evaluation step we quantified the unique peptides with single and double amino acid substitutions (HC T35, HC T38, HC T13, HC T12, LC T4) relative to their corresponding reference peptides (an example is shown in Figure S1, heavy chain (HC), light chain (LC)). The mean calculated relative amount of the five variant peptides relative to their reference peptides measured in quadruplicate was 1.8±0.1%. This result is in good agreement to the spiked level of 1% taking into account the low concentration of the spiked peptide and the potentially different ionization properties of the peptide pairs containing amino acid exchanges such as Glu → Gly or Asp → Trp.

**Table 3:** Quantitation of phenylalanine substitution at the most prominent substitution site in the rhumAb C (HC T36) under different phenylalanine feeding conditions in 2 L fermentation runs.

Phe supplementation (supp)	c(Phe, day 12) [mM]	c(Phe, day 14) [mM]	Phe→Tyr* [%]	Phe→Leu/Ile [%]
“no Phe”	0.03	0.00	0.5	0.1
“low supp Phe”	0.35	0.00	0.3	<0.1
“medium supp Phe”	0.93	0.55	<0.1	<0.1
“high supp Phe”	2.26	2.89	<0.1	<0.1

* :The phenylalanine substitutions were quantified at peptide level in the harvested product (day 14) by adding the areas of the respective peak pair and dividing by the sum of the areas of the all three peaks, the native and the two altered peptide peaks. A quantification at the level of the intact or reduced protein was not possible due to lack of sensitivity and due to other potential isobaric modifications with a mass delta of +16 Da.

#### Case study 1: Detection of low level sequence variants and confirmation by bidirectional ultra-deep DNA sequencing

The described approach was applied to the sequence variant analysis of two antibody batches originating from different cell clones. As for the spiked sample, no differences could be detected in the overlay of the TICs or UV chromatograms. However, differences became visible in the SIEVE derived scatter plot after chromatographic alignment (Figure 4).

Applying the new approach, differences in the glycosylation pattern, in the processing of the light chain signal peptide but also two single amino acid mutations in the light and in the heavy chain of the recombinant antibody samples were detected (Table 2). The quantitation of two sequence variants relative to their corresponding reference peptide revealed a sub-percentage but constant level for the LC sequence variant in both, the 2 L and 100 L fermentation scale and a low percentage level for the HC sequence variant with a slight tendency to increase with fermentation scale.

**Figure 5:**
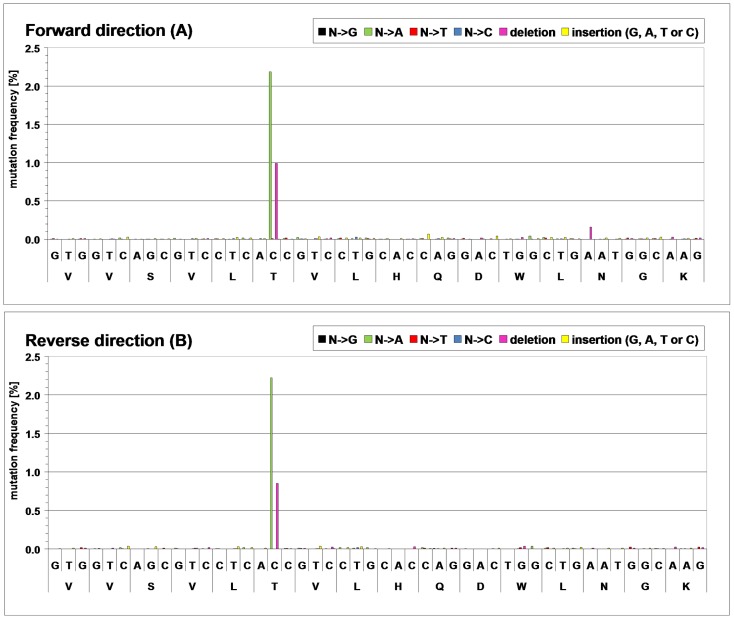
Frequency of base exchanges, deletions or insertions within the 48 base pair DNA region coding for HC peptide VVSVLTVLHQDWLNGK as it was detected by bidirectional ultra-deep sequencing. Separate statistics are shown for base substitutions by guanine (N→G), adenine (N→A), thymine (N→T) or cytosine (N→C). Upper panel (A): sequencing in forward direction; lower panel (B): sequencing in reverse direction.

We assumed that the identified low level sequence variant Thr → Asn may have occurred through a single base substitution at DNA level rather than mistranslation. This offered us the opportunity to confirm this sequence variant by other means and to validate our method. For this purpose, we analyzed the DNA region coding for the affected peptide by ultra-deep DNA sequencing applying the 454 sequencing technology [19]. Both strands of the 48 base pair region were sequenced. Approximately 200,000 valid full-length reads were obtained in each direction (100%). Bioinformatic analysis of the mass sequence data revealed that 2.19% of the forward reads and 2.22% or the reverse reads displayed an adenine instead of a cytosine at the second position of the suspected threonine codon which thereby changed to an asparagine codon (see Figure 5).

The frequency of this cytosine to adenine base mutation corresponds to the frequency of the threonine to asparagine variant observed by LC-MS/MS. We concluded that the threonine to asparagine variant originates from a single point mutation at DNA level. Interestingly, the same cytosine was found to be deleted in 0.90% of the forward reads and in 0.85% of the reverse reads. As a consequence, a frame shift occurred during translation that caused ribosomes to stop at a non-sense codon located 21 nucleotides downstream. Other base exchanges, insertions or deletions were displayed with individual frequencies below 0.10%, with exception of one deletion at 0.16%. However, this deletion was only displayed by forward sequencing but not by reverse sequencing.

**Figure 6:**
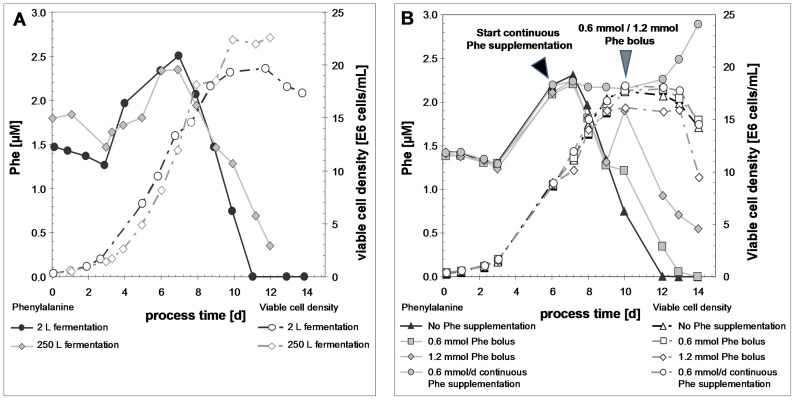
Time course analysis of phenylalanine concentration and viable cell density during fermentation runs of a cell line expressing rhumAb C. A: Comparison of fed-batch (2 L) and scale-up (250 L) fermentation runs without phenylalanine supplementation. B: Comparison of fed-batch fermentation runs (2 L) under different phenylalanine nutrition regimes.

#### Case 2: Low level sequence variant analysis of rhumAb C batches originating from CHO cell fed-batch culture under varying media conditions

In this case the impact of the phenylalanine concentration in the feeding medium on its substitution at protein level was tested. The study resulted from the observation that several production cell clones originating from independently transfected cells showed multiple but identical sites of amino acid sequence changes from phenylalanine to either tyrosine or leucine/isoleucine. We therefore concluded that the observed sequence variants resulted from mistranslation rather than a genetic mutation (data not shown). The samples were obtained after a 14 day 2 L scale fermentation process in fed-batch mode with a commercially available system consisting of chemical defined, protein- and hydrolysate-free basal and feed media. The viable cell density increased to ∼180–220×10^5^ viable cells/mL at day 10 when the cells entered the stationary phase (Figure 6, A). A time-course analysis of the phenylalanine concentration in the cell culture supernatant revealed a slight decrease during the first days, then an increase after the first cell feed between day 3 and 7 and subsequently a rapid decrease below the limit of detection at day 10/11 (Figure 6, A). The fermentation course was reproduced in two other 2 L fermentation runs (data not shown) but was slightly different at 250 L fermentation scale. Having a one day extended cell growth lag phase in the scale-up fermentation, the drop of the phenylalanine concentration after day 7 was not as pronounced as in the 2 L fermentation scale even though the viable cell density reached slightly higher levels (Figure 6, A). A minimal phenylalanine concentration of 0.35 mM in the cell culture supernatant at day 12 was reached at harvest.

The amino acid substitutions Phe → Tyr and Phe → Leu/Ile were detected for the product obtained from the fed-batch fermentation runs but not the scale-up 250 L fermentation, where the Phe concentration was never below 350 µM. Whereas the Phe → Leu/Ile substitution occurred only at one position in the protein and was always found at lower levels than the Phe → Tyr substitution, the later was found in both antibody chains at almost all phenylalanine positions at levels between 0.3–0.6% (Table S1). When applying the described sequence analysis approach and analyzing the data using Mascot ETS with the original unimod.xml modification file, the database search assigned the altered peptides to peptides oxidized at the phenylalanine residues as top ranking hit. The phenylalanine to tyrosine substitution was always the second hit for the respective exact mass and MS/MS spectra even though the ion score for both, the modification and the mutation was identical.

**Figure 7:**
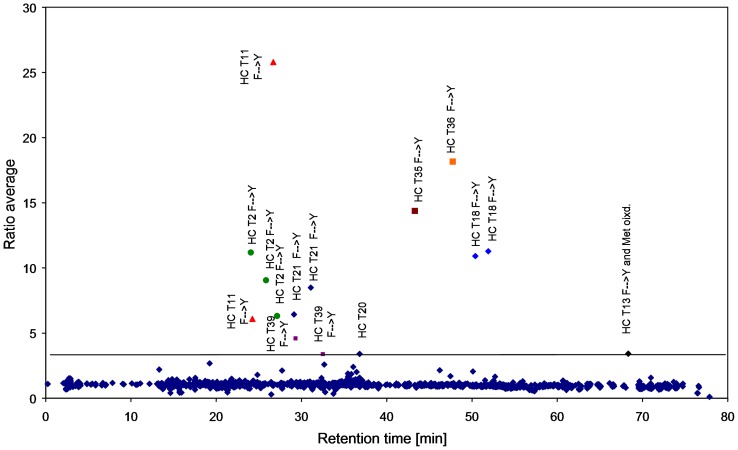
Differential analysis of mass/retention time profiles of two LC-MS/MS data set replicates from tryptic digests of recombinant human IgG1 antibody C under phenylalanine starvation versus phenylalanine supplementation conditions. The horizontal line indicates the minimal intensity ratio value above which m/z signals are considered. Peaks showing identical MS and MS/MS data but eluting at different retentions time are marked by shape and color.

If the absence of phenylalanine was the root cause of the observed phenylalanine substitutions it should not appear when supplementing this amino acid. Therefore, in different 2 L fed-batch experiments phenylalanine was supplemented in different concentrations (low, medium and high) to feed media according specific Phe consumption rate calculated in initial experiments. Differences in cell growth and productivity performance were not detected. A time-course study of the extracellular phenylalanine concentration was conducted (Figure 6, B) showing that the “medium supp Phe” condition prevents phenylalanine to go into limitation. The correlation of the phenylalanine substitution to its supplementation is exemplified using the most prominent substitution site (HC T36) (Table 3). The data in the table indicate that indeed the substitution of phenylalanine can be prevented by supplementation.

Interestingly, when applying the differential analysis of the mass/retention time profiles of the antibody batch from fermentation without phenylalanine supplementation with the corresponding batch with the highest phenylalanine supplementation, it became clear that the phenylalanine to tyrosine substituted peptides always appeared as peak pairs in the retention time domain (Figure 7).

Those altered peptides containing more than one phenylalanine such as HC T3 appeared even at three retention times. For some altered peptides (HC T21, T36 and T37), the second peak yielded a relatively low ratio and is therefore not labeled in Figure 7. Peptides HC T40 and T14 contained more than one phenylalanine but the site of substitution could not be identified from the MS/MS spectrum. The reason for the elution at two retention times is unclear up to now. Other peptides containing tyrosine in their native sequence have been evaluated for their retention time behavior and were not found to elute at two different retention times. The possibility that one of the peaks could be oxidized phenylalanine has been ruled out as the reference sample obtained from the phenylalanine supplemented fermentation did not give any hint towards oxidized phenylalanine. The double retention time phenomenon was not observed for other amino acid substitutions so far and even phenylalanine to leucine/isoleucine did not result in a retention time peak pair.

## Discussion

The detection and identification of protein sequence variants is commonly achieved using relatively laborious methods such as radiolabelling techniques [20], [21], amino acid analysis [20], [9], N-terminal sequencing of peptide fractions [9] or peptide mapping with reversed phase HPLC-MS [14]. These techniques are limited to the analysis of particular amino acid substitutions [17], [20] and/or to the number of samples that can be analyzed within a reasonable time. Furthermore, they often lack site specific information. The analytical two-step approach presented here overcomes these limitations. A multi sample comparison provides quantitative sequence variant information in a very fast manner as a first step. For this, at least two protein samples from independent experiments are required, e.g. from different transfections, cell ages or fermentation media. Samples without any detectable deviation from the used reference can be sorted out immediately. Samples with detectable deviations need to be subjected to full characterization including identification, verification and quantitation of aberrant sequence variants as a second stage.

The knowledge gained by full characterization is not restricted to the presence or absence of single point mutations but also comprises the identification of other aberrant variants such as double mutations, peptide elongations or potential amino acid isomerization. Sequence variants can be detected at peptide level with sensitivity as low as 1%, some of them even down to 0.1%. However, even when applying these optimized conditions some sequence variants might be missed especially in those parts of the protein that yield very small or very large tryptic peptides. In order to cover these parts of the analyte, a second enzymatic digest can be performed.

The examples shown reflect the experience gained with the sequence variant analysis in our laboratories. Case 1 shows the strength of the “quantitative” comparison of batches, as low level differences were identified, DNA point mutations as well as an incorrectly processed signal peptide. This exemplifies that the root cause of protein variants can be manifold. Although alternate cleavage of signal peptides has been reported in the literature [22], the signal peptide remaining shown here has not been reported up to now and has a relatively low probability when predicted by published methods [23]. Ultra-deep DNA sequencing was found to be suitable for the confirmation of a rare point mutation that was assumed after sequence variant analysis at the protein level. In contrast to mutation specific qPCR, which has been used to detect DNA sequence variants in recombinant cell lines [10] and, in general, allows quantitative detection of single nucleotide variations down to 0.1% [24], ultra-deep sequencing does not require the design of specific primers or probes nor the set-up of selective PCR conditions. In the example reported here, we were able to address a single amino acid substitution that occurred at 2% level, to a single base substitution that was displayed with similar incidence. Due to the low level of background signals which are generally below 0.1%, we suppose that point mutations can be identified reliably down to 0.5%. Additional experiments need to be done to test if ultra-deep sequencing is also suitable for the *de novo* detection of sequence variants caused by DNA mutations. This would require sequencing of complete genes and comparative analysis of LC-MS/MS and DNA sequencing data.

The second case study shows that cells misincorporate tyrosine at phenylalanine positions when starvation of phenylalanine occurs. Characteristic for the phenylalanine to tyrosine substitution is that the variant peptides appear at two different retention times. One hypothesis for explanation is that the fermentation media contains not only *para* but also *meta* or *ortho* tyrosine leading to the misincorporation of tyrosine isomers. The mischarging of tRNA^Phe^ with *meta*-Tyrosine has been reported in the literature [25]. The level of misincorporation is in the sub-percentage range (see Table 3) but in contrast to genetically based sequence variants occurs at multiple non-codon specific sites and can be eliminated by phenylalanine supplementation.

To summarize, the developed approach can detect sequence variants and differences between samples of manifold origin and nature in a sensitive and more comprehensive manner. It will improve the developmental process of recombinant biotherapeutics but it may also help to elucidate the mechanisms of misincorporation of amino acids and the structure-function relationship between sequence variants and biological impact.

## Materials and Methods

### Antibodies and Chemical Reagents

Recombinant human monoclonal antibodies (rhumAbs) A (immunoglobuline G 1 (IgG1)) and B (IgG4) were expressed in stably transfected CHO cell lines (CHO-K1 ATCC # CCL-61T). Recombinant human monoclonal antibody (rhumAb) C (IgG1) was expressed in stably transfected CHO cell line (CHO-K1SV, Lonza Biologics, Basel, Switzerland). Standard chemical reagents were used for analysis.

### Bioreactor Cultures, Cell Growth and Amino Acid Analysis and Product Purification

CHO clones were grown in 2L Quad fermentation systems, in 100 L and 250 L bioreactor according to feeding conditions described in the supporting material section. Cell growth and viability were analyzed by using the trypan blue exclusion method [26] and an automated CedexHiRes device (Roche Innovatis, Bielefeld, Germany). Amino acids were analyzed according to a protocol described by Agilent Technologies [27] using a rapid resolution HPLC system (Agilent 1200, Agilent Technologies Inc., Waldbronn, Germany). The harvested rhumAbs were purified from 2 L fermentation experiments by small scale Protein A HPLC method and from 100 L and 250 L fermentation experiments by a multistep chromatographic procedure. The purity of the samples was >90% as determined by size exclusion chromatography.

### Tryptic Digestion and On-line RP-LC-MS/MS

Antibody samples were denatured, reduced alkylated, buffer exchanged and trypsin digested. For preparation of the spiking sample, a 1% (v/v) of a tryptic digest of rhumAb B was mixed with a tryptic digest of rhumAb A. The peptide mixture obtained was injected and separated without pretreatment using reversed phase HPLC (Agilent 1100 Cap LC, Agilent Technologies, Böblingen, Germany). A Varian Polaris 3 C18 – Ether column (1×250 mm; 3 µm particle diameter, 180 Å pore size) from Varian (Darmstadt, Germany) was used for separation. The HPLC eluate was split using Triversa NanoMate (Advion, Ithaca, NY, USA) and 380 nL/min were infused into a LTQ Orbitrap classic tandem mass spectrometer (Thermo Fisher Scientific, Dreieich, Germany) operating in positive ion mode.

### Ultra Deep DNA Sequencing

Ultra-deep DNA sequencing was performed using the pyrosequencing technology of 454 Life Sciences (Branford, Connecticut).

A detailed description of all materials and protocols used can be found **Text S1**.

## Supporting Information

Figure S1 **Extracted ion chromatograms of two tryptic peptides (T HC36 rhumAb A and HC T35 rhumAb B) differing in one amino acid in the reference versus the 1% spiked antibody.** Quantification by EIC results in 1.65±0.06% for n = 4 LC runs of spiked peptide versus reference peptide.
(TIF)

Figure S2 **MS/MS spectra of the variant peptide (A) and the expected peptide (B) of rhumAb A from clone 1 detected by with the SIEVE scatter plot and identified by Mascot ETS.**
(TIF)

Figure S3 **MS/MS spectrum of a variant peptide detected by SIEVE analysis and identified using sequence tags of the known antibody sequence together with the knowledge of the signal peptide used for expression of the antibody light chain.**
(TIF)

Table S1 **Quantitation of Phe → Tyr substitution at different substitution sites in the sequence of rhumAb C under phenylalanine starvation conditions.**
(DOC)

Text S1 **Detailed description of materials and methods.**
(DOC)
