# Supplementary figures and images for: Low Level Sequence Variant Analysis of Recombinant Proteins: An Optimized Approach
Source: PLoS One. 2012 Jul 6;7(7):e40328. doi: 10.1371/journal.pone.0040328 (PMC3391300; doi:10.1371/journal.pone.0040328)

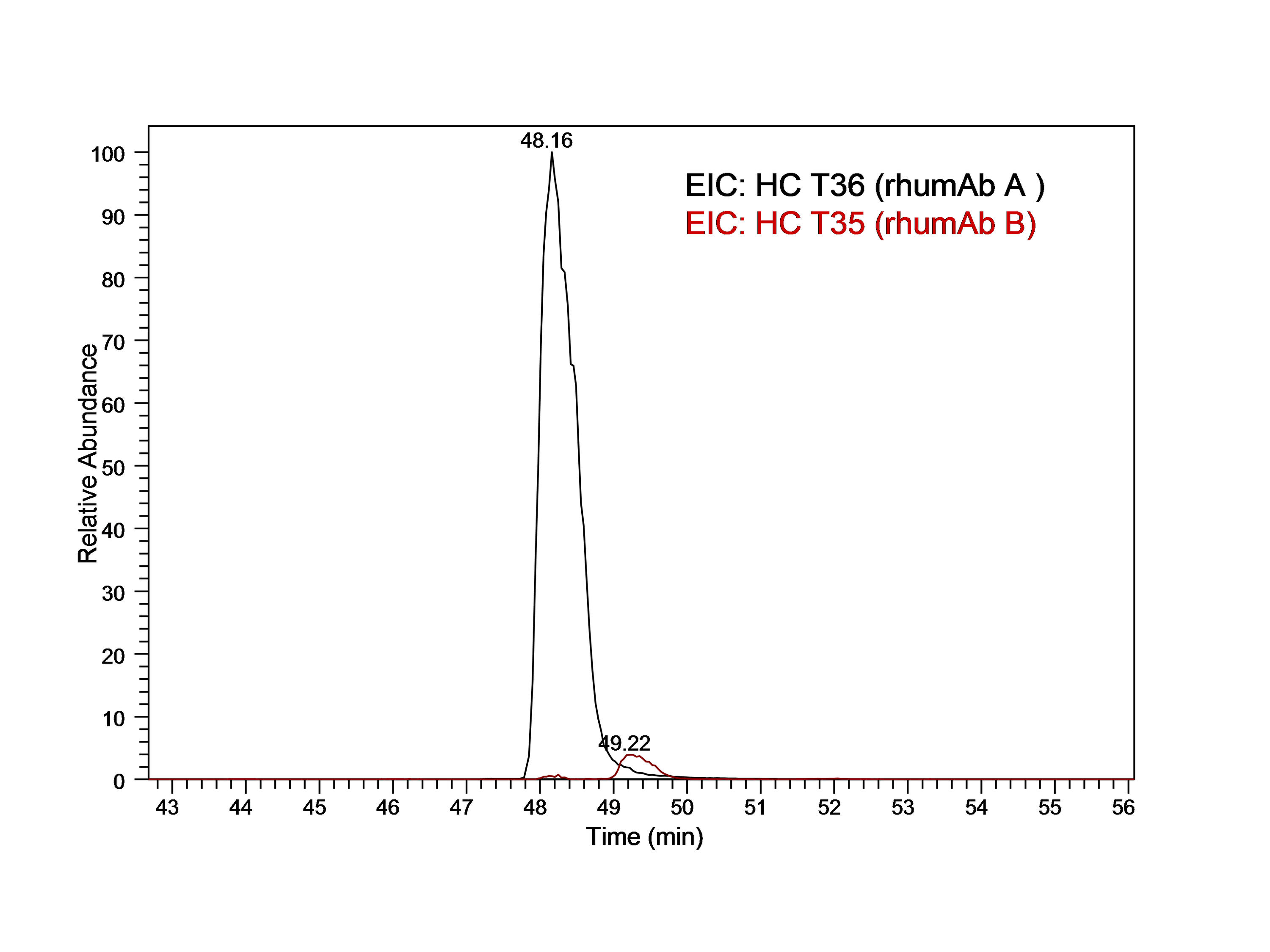

Supplement: Figure S1 — Extracted ion chromatograms of two tryptic peptides (T HC36 rhumAb A and HC T35 rhumAb B) differing in one amino acid in the reference versus the 1% spiked antibody. Quantification by EIC results in 1.65±0.06% for n = 4 LC runs of spiked peptide versus reference peptide. (TIF) [file pone.0040328.s001.tif]

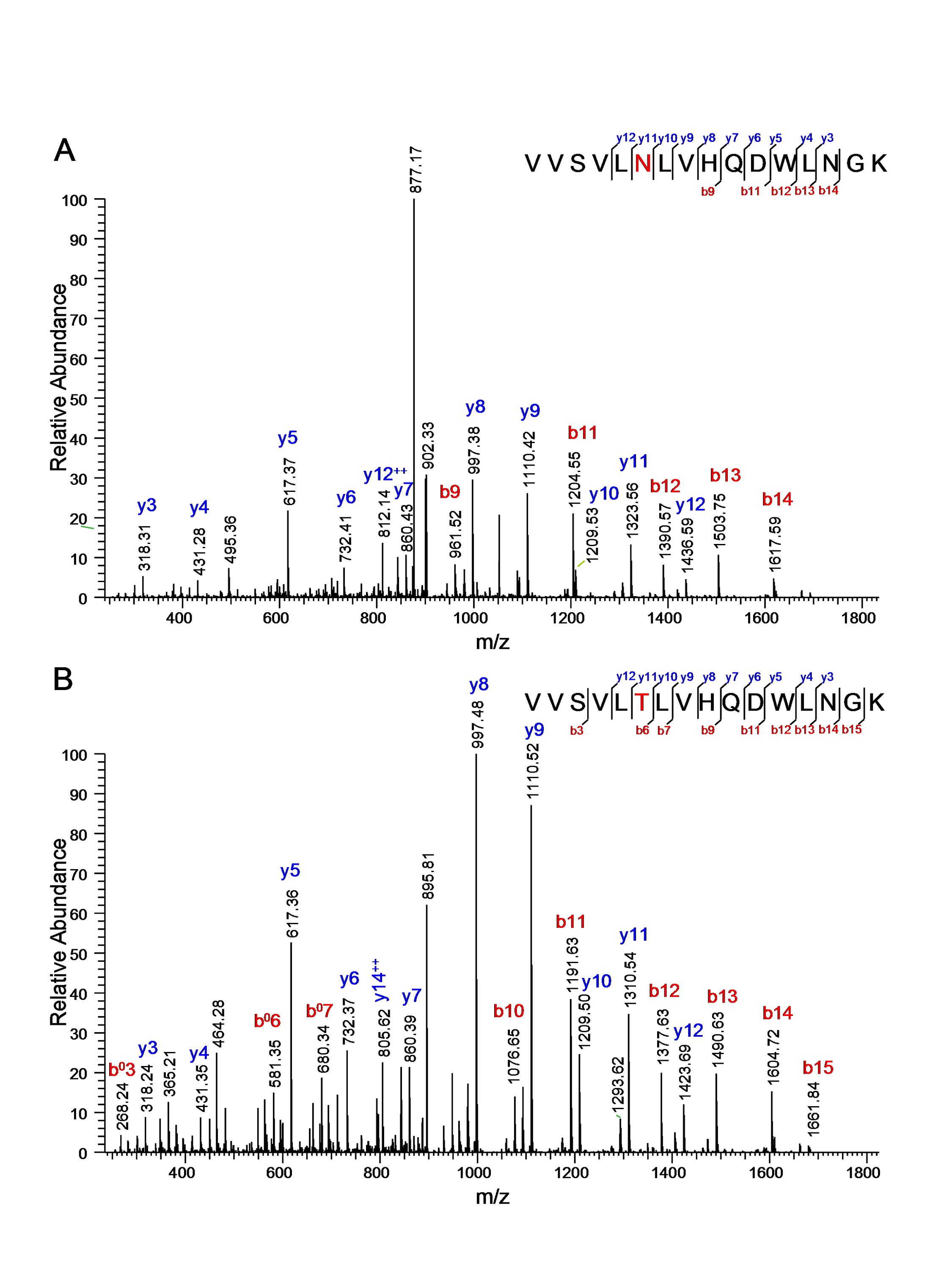

Supplement: Figure S2 — MS/MS spectra of the variant peptide (A) and the expected peptide (B) of rhumAb A from clone 1 detected by with the SIEVE scatter plot and identified by Mascot ETS. (TIF) [file pone.0040328.s002.tif]

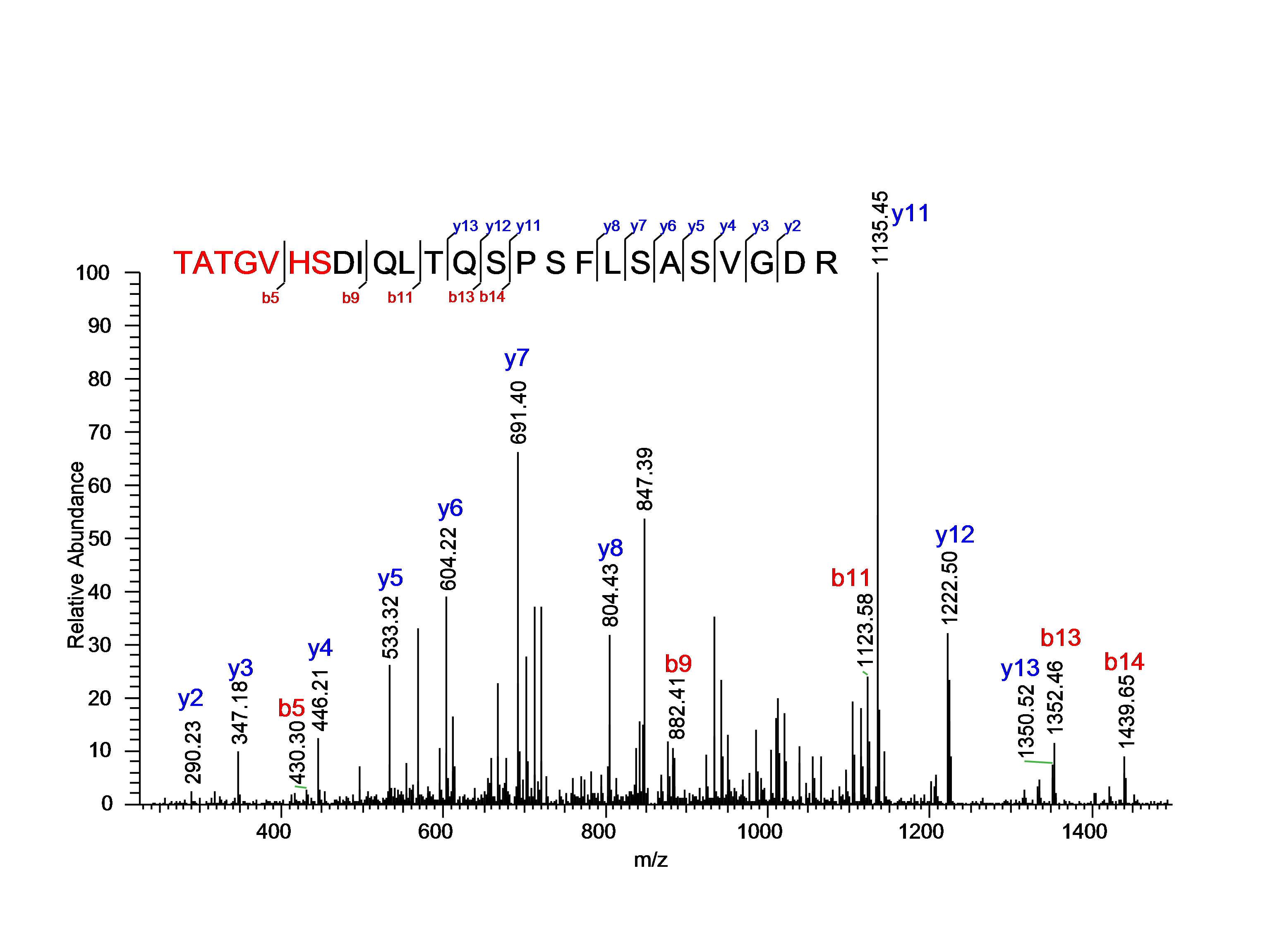

Supplement: Figure S3 — MS/MS spectrum of a variant peptide detected by SIEVE analysis and identified using sequence tags of the known antibody sequence together with the knowledge of the signal peptide used for expression of the antibody light chain. (TIF) [file pone.0040328.s003.tif]
