# Supplementary material for: Low Level Sequence Variant Analysis of Recombinant Proteins: An Optimized Approach
Source: PLoS One. 2012 Jul 6;7(7):e40328. doi: 10.1371/journal.pone.0040328 (PMC3391300; doi:10.1371/journal.pone.0040328)
Supplement: Table S1 — Quantitation of Phe → Tyr substitution at different substitution sites in the sequence of rhumAb C under phenylalanine starvation conditions. (DOC) [file pone.0040328.s004.doc]

**Table S-1.** Quantitation of Phe 🡪 Tyr substitution at different substitution sites in the sequence of rhumAb C under phenylalanine starvation conditions.

| HC T# | RT1 | % Peak 1 | RT2 | % Peak 2 | % total SV |
| --- | --- | --- | --- | --- | --- |
| HC T2 | 25.7 | 0.1 | 26.9 | 0.2 | 0.3 |
| HC T6 | 18.4 | 0.1 | 19.2 | 0.2 | 0.3 |
| HC T11 | 24.2 | 0.1 | 26.6 | 0.3 | 0.4 |
| HC T13 * | 66.2 | 0.2 | 68.3 | 0.6 | 0.8* |
| HC T18 | 50.4 | 0.2 | 52 | 0.2 | 0.4 |
| HC T21 | 29 | 0.1 | 31 | 0.3 | 0.4 |
| HC T35 | 41.3 | 0.1 | 43.5 | 0.2 | 0.3 |
| HC T36 | 39.8-43 | 0.2 | 47.5 | 0.3 | 0.5 |
| HC T39 | 29.3 | 0.1 | 32.3 | 0.1 | 0.2 |

*HC T13 variant is a mixture of methionine oxidation and phenylalanine 🡪 tyrosine substitution.
