# Supplementary material for: Low Level Sequence Variant Analysis of Recombinant Proteins: An Optimized Approach
Source: PLoS One. 2012 Jul 6;7(7):e40328. doi: 10.1371/journal.pone.0040328 (PMC3391300; doi:10.1371/journal.pone.0040328)
Supplement: Text S1 — Detailed description of materials and methods. (DOC) [file pone.0040328.s005.doc]

Supporting Information

Low Level Sequence Variant Analysis of Recombinant Proteins: an Optimized Approach

Anne Zeck^1,3^, Jörg Thomas Regula^1^, Vincent Larraillet^1^, Björn Mautz^1^, Oliver Popp^1^, Ulrich Göpfert^1^, Frank Wiegeshoff^1,2^, Ulrike E.E. Vollertsen^1^, Ingo H. Gorr^1^, Hans Koll^1^, Apollon Papadimitriou^1^

^1^Biologics Research, Pharma Research and Early Development (pRED)

Roche Diagnostics GmbH

Penzberg

Germany

^2^ present address after completion of the study

Abbott GmbH

Ludwigshafen

Germany

* to whom correspondence should be addressed: anne.zeck@nmi.de

CORRESPONDING AUTHOR FOOTNOTE. ^3^ present address after completion of the study: NMI Natural and Medical Sciences, Institute at the University of Tuebingen, Reutlingen, Germany.

### **Materials and Methods**

Materials

*Cell lines and antibodies*. Recombinant human monoclonal antibodies (rhumAbs) A (IgG1), B (IgG4) or C (IgG1) were expressed in stably transfected CHO cell lines. These were propagated in serum-free media at 37 °C and 5-8 % CO_2_ under humidified conditions and constant agitation. For stable cell line generation, CHO suspension cells had been transfected with a plasmid vector coding for the antibody heavy chain (HC) and light chain (LC). Transfected cells were selected in multi-well plates and subcloning was performed by limiting dilution. Production cell line candidates were identified based on antibody expression and growth properties.

*Bioreactor cultures, cell growth and amino acid analytics and product purification*

CHO clones were grown in 2 L Quad fermentation systems, in 100 L bioreactor (both Sartorius Stedim Biotech, Aubagne Cedex, France) and/or 250 L Hyclone single use bioreactor systems (SUB) (Thermo Scientific, Erembodegem, Belgium). For fed-batch cultivations of CHO clones, rhumAb A and rhumAb B expressing cells were fed continuously starting from 48 h until 72 h with a feed rate of 3 % (v/v) of start volume/day and from 72 h until harvest with 6 % (v/v) of start volume/day. For fed-batch cultivations of the CHO clone expressing rhumAb C cells were fed by two continuous feeds. The first feed was given with a rate of 2 % (v/v) of start volume/day for the first four days until the viable cell density reached 20 x 10^5^ cells/ml. The second feed started subsequently and was given until the end of fermentation with a rate of 1 % (v/v) of start volume/day. Cell growth and viability were analyzed by using the trypan blue exclusion method^22^ and an automated CedexHiRes device (Roche Innovatis, Bielefeld, Germany). Amino acids were analyzed according to a protocol described by Agilent Technologies using a rapid resolution HPLC system (Agilent 1200, Agilent Technologies Inc., Waldbronn, Germany).

The harvested rhumAbs were purified from 2 L fermentation experiments by small scale Protein A HPLC method and from 100 L and 250 L fermentation experiments by a multistep chromatographic procedure.

*Chemical reagents*

Tris(hydroxymethyl)aminomethane (TRIS Base), iodoacetic acid sodium salt, formic acid, hydrochloric acid and sodium chloride were obtained from Merck (Darmstadt, Germany). Dithiothreitol (DTT), was obtained from Roche Diagnostics (Mannheim, Germany). Guanidinium hydrochloride, HPLC-grade water with 0.1% formic acid, HPLC-grade acetonitrile with 0.1% formic acid was obtained from Fluka (Munich, Germany). Sequencing-grade trypsin, was purchased from Promega Corporation (Mannheim, Germany).

*Tryptic digestion and on-line reversed phase chromatography tandem mass spectrometry (LC-MS/MS)*

The sample preparation part was optimized with regard to reproducibility (e.g. by avoidance of chymotryptic cleavage or K/P cleavage) and the avoidance of sample preparation artifacts (e.g. deamidation, oxidation). Antibody samples were denatured and reduced by mixing 50 µL of rhumAbs (5 mg/mL) with 240 µL of denaturing buffer (0.4 M Tris/HCl, 8.0 M Gua-HCl, pH 8.0) and 20 µL of 0.24 M freshly prepared DTT. The reduction was carried out at 37 °C for 1 hour. The reduced samples were cooled at room temperature and subsequently alkylated by addition of 20 µL freshly prepared alkylation reagent (0.6 M iodoacetic acid). The alkylation process was carried out at room temperature in the dark for 15 minutes. The excess of alkylation reagent was inactivated by addition of 30 µL of DTT solution. The samples were than buffer exchanged to approx. 480 µL of 50 mM Tris/HCl, pH 7.5 using Sephadex G-25 DNA grade columns (GE Healthcare Bio-Sciences AB, Uppsala, Sweden). Digestion was performed with trypsin for 2.5 h at 37 °C at an enzyme to substrate ratio of 72:1 (w/w) and addition of another aliquot of trypsin to obtain an enzyme to substrate ratio of 36:1 for another 2.5 h at 37 °C. The digestion was stopped by addition of 20 µL 10 % formic acid. The digested samples were stored at ≦ – 20 °C until injection onto the column.

For preparation of the spiking sample, a 1 % (v/v) of a tryptic digest of rhumAb B was mixed with a tryptic digest of rhumAb A. The peptide mixture obtained was injected and separated without pretreatment using reversed phase HPLC (Agilent 1100 Cap LC, Agilent Technologies, Böblingen, Germany). A Varian Polaris 3 C18 – Ether column (1 x 250 mm; 3µm particle diameter, 180 Å pore size) from Varian (Darmstadt, Germany) was used for separation. An optimized 105-minute linear gradient with varying slopes was applied at 37 °C as follows (minute/%B): 0/2, 5/2, 15/15, 70/32, 80/38, 85/100 90/100 92/2 100/2. The sample injection was 20 µL (~ 4.7 µg). The HPLC eluate was split to a ratio of 1:150 using Triversa NanoMate (Advion, Ithaca, NY, USA). 380 nL/min were infused into a LTQ Orbitrap classic tandem mass spectrometer (Thermo Fisher Scientific, Dreieich, Germany) operating in positive ion mode. A detailed description of the parameters used for data acquisition and processing can be found in the supporting information.

*Parameter used for data acquisition and processing*

The data acquisition part was optimized with regard to retention time stability and the reproducible triggering of as many as possible high quality fragment ion spectra. All samples were measured in duplicates. The NanoMate spray voltage was 1.65 kV, the capillary temperature was 185 °C and the tube lens voltage was 100 V. For the MS/MS product ion scan, the activation type was collision-induced dissociation (CID) with stepped collision energy enabled with collision energy width of 20 in 5 steps. The 5-scan-event LTQ Orbitrap method applied consists of a MS scan at m/z 80-800 with source induced dissociation set to 92 V and resolution power (RP) of 15.000 and a full MS survey scan (m/z 350-2000) at RP 60.000 followed by three cycles of data-dependent MS/MS scans on the top three most intense ions. The dynamic exclusion function was enabled and parameters were as follows: a repeat count of 2, a repeat duration of 20 sec, exclusion duration of 30 sec, an exclusion list size of 500, an exclusion mass width of 5 ppm. Unassigned charge states were rejected for MS/MS triggering and a reject mass list containing polysiloxan ions [1] was enabled. MS/MS data were grouped using a precursor ion tolerance of 10 ppm and a group scan number of 50 using Bioworks 3.1. For data processing the Mascot 2.3 error tolerant search (Matrix Science London, UK) based on an in house protein database was used. The data base contained the sequences of all antibodies under development in the company as well as the sequences of the used proteolytic enzymes. Data base search was performed using 8 ppm peptide mass tolerance. No minimal ion score cut-off was used as its determination by the software was unclear and seemed to vary depending on the Mascot server version. All potential sequence variants were manually inspected taking into account their isotopic pattern and their retention time behavior as well as their MS/MS fragment spectrum in comparison to the reference peptide. SIEVE 1.3 (Thermo Fisher Scientific, Dreieich, Germany) was used for comparative analysis of data sets. LC-MS/MS data sets were acquired in replicates and chromatographic alignment was performed using two replicates of both, the control and the sample data set. SIEVE framing parameters were as follows: a retention time width of 2 min, m/z width of 1.6 amu, a search peak width of 30 % and a signal threshold of 100.000. A cut-off ratio value of 2.5 for signals considered different was defined based on the optimized acquisition and framing parameters.

For relative quantification, the extracted ion chromatograms were calculated using all charge states and all isotopes. The peak areas were determined by integration and the relative sequence variant content was calculated by dividing the peak area of the peptide variant by the sum of the peak areas of the reference and the variant peptide.

*Ultra deep DNA sequencing*

Ultra-deep DNA sequencing was performed using the pyrosequencing technology of 454 Life Sciences (Branford, Connecticut). Genomic DNA was prepared from the rhumAb A producing CHO cell line using the Genonic DNA Kit (Qiagen, Hilden, Germany) according to the manufacturers protocol. A 215 base pair fragment of CH_2_ coding region was amplified by PCR employing *Pfu* polymerase (Promega, Madison, Wisconsin) and fusion primers that consist of a 23-24 base pair target gene specific sequence at the 3’-end and a fixed 19 base pair sequence (A or B) at the 5’ end. Fusion primers A_for (5’- GCCTCCCTCGCGCCATCAGcgaagaccctgaggtcaagttca-3’) and B_rev (5’-GCCTTGCCAGCCCGCTCAGtggctttggagatggttttctcga-3’) were used for sequencing of the forward strand, fusion primers A_rev (5’-GCCTCCCTCGCGCCATCAGtggctttggagatggttttctcga-3’) and B_for (5’- GCCTTGCCAGCCCGCTCAGcgaagaccctgaggtcaagttca-3’) were used for sequencing of the reverse strand. The amplicons were purified by agarose gel electrophoresis and extracted from the gel using the QIAquick Gel Extraction Kit (Qiagen, Hilden, Germany). Clonal amplification, sequencing on Genome Sequencer FLX system and biostatistical analysis were performed at Eurofins MWG (Ebersberg, Germany).

*Reference:*

[1] Schlosser A, Volkmer-Engert R (2003) Volatile polydimethylcyclosiloxanes in the ambient laboratory air identified as source of extreme background signals in nanoelectrospray mass spectrometry. J Mass Spectrom 38: 523-525.
